# Early Clinical Experience with Silver-Ion Doped Synthetic Bone Grafts for the Treatment of Chronic Bone Infections: A Retrospective Study

**DOI:** 10.3390/jcm15010029

**Published:** 2025-12-20

**Authors:** Bünyamin Yücel, Aydan Ayşe Köse, Nusret Köse

**Affiliations:** 1 Department of Orthopedics and Traumatology, Faculty of Medicine, Eskisehir Osmangazi University, Eskişehir 26040, Turkey; drbunyaminyucel@gmail.com; 2 Department of Plastic and Reconstructive Surgery, Faculty of Medicine, Eskisehir Osmangazi University, Eskişehir 26040, Turkey; aydan.kose@gmail.com

**Keywords:** chronic osteomyelitis, silver ion-doped graft, calcium phosphate, antimicrobial bone substitute, infection eradication

## Abstract

**Background/Objectives**: Chronic bone infections require local antimicrobial delivery to achieve high drug concentrations while limiting systemic toxicity. Silver ion-doped calcium phosphate synthetic bone grafts have been proposed as carriers for local antimicrobial release. This study aimed to evaluate the efficacy and safety of a silver ion-doped synthetic bone graft in patients with chronic osteomyelitis, infected nonunion, or implant-related bone infection. **Methods**: This retrospective cohort included 12 adults who underwent surgery for chronic osteomyelitis or implant-associated infection. All patients received thorough debridement, removal of infected implants when present, and filling of bone defects with a silver ion-doped calcium phosphate graft. The median age was 38 years, and follow-up was 12 months. Clinical and radiographic outcomes, liver and kidney function tests, and blood silver levels were assessed pre- and postoperatively. **Results**: Infection eradication was achieved in 11 of 12 patients (90%) at 12 months. Functional recovery, defined as return to normal daily activities, occurred within 3–5 months. Bone union was observed in all but one patient within 3–6 months, and no graft resorption was detected at one year. No significant differences in liver or kidney function tests were found compared with the control group (*p* > 0.05), and blood silver levels remained within normal limits. **Conclusions**: At 12-month follow-up, silver ion-doped synthetic bone grafts showed encouraging safety and efficacy in the treatment of chronic osteomyelitis. These findings suggest that silver-doped grafts may represent a useful option for one-stage treatment of osteomyelitis.

## 1. Introduction

Bone infection, commonly referred to as osteomyelitis, is typically categorized into acute and chronic forms. Acute osteomyelitis can often be successfully treated with antibiotics when diagnosed and managed promptly. In contrast, chronic osteomyelitis is much harder to eradicate, as it involves an ongoing inflammatory process that causes progressive bone damage and the formation of sequestra. The incidence of chronic osteomyelitis is increasing because of the higher frequency of disorders that lead to infection, such as obesity, diabetes mellitus, malignancy, immune deficiencies, trauma, and orthopedic surgeries. In orthopedic practice, chronic osteomyelitis remains one of the most challenging problems faced by orthopedic surgeons due to the long-term morbidity and even increased risk of death. It represents a heavy economic burden for patients and society [1,2].

Successful treatment of bone infection often requires local delivery devices made of absorbable or non-absorbable materials that can provide long-term high concentrations of antibiotics locally, along with adequate surgical debridement, which may often be achieved with a wide excision [3]. For several years, bone defects in osteomyelitis have been treated through debridement in combination with gentamicin-loaded poly-methyl methacrylate. However, this method is not free of drawbacks, such as thermal damage to the antibiotic, the need for a second intervention to remove PMMA, and facilitation of biofilm formation by multiresistant colonies due to inadequate antibiotic concentration. To address the drawbacks of antibiotic-loaded poly-methyl methacrylate, biodegradable synthetic bone graft substitutes have been developed [4]. These biomaterials, which have chemical and crystal properties similar to those of bone, may promote osteoconduction and osteogenesis and have been used to repair bone defects. Bone substitutes impregnated with antibiotics can release locally therapeutic amounts of antibiotics over long periods [2,5].

In the treatment of bone infections, traditional methods that can provide the minimum inhibitory concentration (MIC) required to eliminate biofilm-forming bacteria locally for extended periods inevitably lead to serious side effects. The development of antibiotic-releasing, bioabsorbable calcium-based scaffolds as an alternative has been known for a long time [6]. Although many clinical studies with positive results regarding these biomaterials have been published in the literature, there are also publications reporting that these studies carry a risk of bias and do not improve the results [7,8]. Despite numerous methods being tested in the treatment of bone infections, the current literature highlights the ongoing challenges in completely eliminating bone infection and achieving excellent bone reconstruction, thus emphasizing the need for advanced and innovative bone replacement materials [9].

The global rise in antibiotic resistance poses a significant threat, diminishing the efficacy of common antibiotics against widespread bacterial infections [10]. To improve bone infection treatment outcomes, there has been growing interest in developing ways to deliver high concentrations of antimicrobials locally while reducing systemic side effects and toxicity. The increase in antibiotic-resistant pathogens leads to consideration of a material-based view of antimicrobials for osteomyelitis [11]. Silver is a metal-based therapeutic that has found widespread use in medicine due to its antimicrobial effects. Numerous published studies have demonstrated that silver, particularly its ionic form, exhibits cytocompatibility, efficacy against planktonic and sessile bacteria, and bactericidal activity against methicillin-susceptible Staphylococcus aureus and methicillin-resistant Staphylococcus aureus (MRSA) strains [12]. Unlike many antibiotics, silver ions exert their effects by activating multiple mechanisms at different targets within bacteria, leading to a lower incidence of clinically resistant bacteria [13].

In our previous studies, a silver ion-doped calcium phosphate-based antimicrobial ceramic coating was developed to increase the biocompatibility of orthopedic implants and prevent bacteria from adhering to implant surfaces [14,15,16]. Since these coatings are effective in preventing implant-related infections, it stands to reason that they might also be effective in treating bone infection when used as a synthetic bone graft. In this context, the efficacy and safety of this antimicrobial graft have been demonstrated microbiologically, radiologically, toxicologically, and histopathologically in animal studies [17].

This study aimed to investigate the clinical efficacy and safety of silver ion-doped calcium phosphate-based synthetic bone graft material as biodegradable substitutes incorporated with antimicrobial function for one-stage treatment of osteomyelitis as an antimicrobial carrier in patients with chronic osteomyelitis.

## 2. Materials and Methods

### 2.1. Study Design and Settings

We performed a retrospective observational study using prospectively collected data on 12 adult patients who had undergone surgery for chronic osteomyelitis, infected nonunion, or implant-related bone infection between 2020 and 2021 at a single tertiary-level referral hospital. Infection was in the tibia in half of the patients (6 cases), in the humerus in two cases, in the hand bones in two cases, and in the femur and calcaneus in one case each. Nine of these cases were postoperative/fracture-related osteomyelitis and three were hematogenous osteomyelitis. The patients gave informed consent for the study. The Ethics in Human Research Committee of the investigating institution gave ethical approval for the study (No: 10-8 June 2016). ClinicalTrials.gov ID NCT03945864. Adult patients who gave consent were entered into the study if they were diagnosed with osteomyelitis, implant-related bone infection, or infected nonunion characterized by clinical symptoms present for greater than 90 days, the presence of necrotic bone, and bacteria cultured from prior procedures, surgical biopsy, or draining sinuses. Patients with compromised immune defense mechanisms and comorbidities such as malignancy, diabetes mellitus, polytrauma, and open fractures, or pregnant individuals, were excluded, as were patients younger than 18 years of age and patients with heart, lung, renal, liver failure, epilepsy, cerebrovascular attack, or ischemia. Patients were excluded if their minimum clinical follow-up was less than 12 months. Eradication of infection, bone healing, and osseointegration of the synthetic bone were accepted as parameters indicating that the treatment result was successful.

### 2.2. Surgical Procedure

Our surgical protocol was based on the surgical debridement of the infected bone area, removal of the infected implant if present, and then filling the resulting bone gaps with silver ion-doped calcium phosphate-based synthetic bone graft. After an open approach with a good overview of the surgical site, multiple deep samples were taken, sinus tracts were excised, and contaminated implants were removed, followed by the excision of necrotic bone, continuing until healthy, bleeding bone was exposed. The area was then irrigated extensively, and the cavity was packed with synthetic bone grafts. No additional material or antibiotic was added, and primary skin closure was achieved directly. Internal or external fixation was applied in cases requiring stabilization. All procedures were performed by the first author (NK). Routine parenteral antibiotic prophylaxis was given to patients during surgery and for the first 24 h (Sefazolin Sodyum 1000 mg, three times a day, IV). All patients were given the standard chronic osteomyelitis postoperative protocol (adjuvant systemic antibiotics parenterally for 1–2 weeks, orally for 6–8 weeks) according to sensitivity tests.

### 2.3. Synthetic Bone Graft Material

Wet chemical synthesis was used to produce silver ion-doped calcium phosphate powders. Ceramic beads with interconnected micro and macro pores were obtained using this powder. These beads contained Hydroxyapatite-Tricalcium phosphate (HAP–TCP). (TRBoneR, TRCoating Inc., Eskişehir, Turkey). In general, Calcium hydroxide is mixed with pure water, and silver nitrate is added to the system, introducing silver ions that provide antimicrobial properties. The pH and stoichiometry of the system are determined using orthophosphoric acid. The resulting precipitate is passed through a filter and dried at 80 °C. The HAP–TCP ratio of the material is determined using pH and temperature. The beads produced had an 80% wt. HAP and a 20% wt. TCP. To ensure the resulting beads have a porous structure (70% porosity) polystyrene space maker balls which act as a fugitive phase was added to the material at a rate of 1% by weight. Then they were dried and sintered at 1200 °C for 1 h to create the macro porous and mechanically strong structure. The synthesis procedure for silver ion-doped calcium phosphates and the results of the silver release and toxicity studies have been reported in previous studies. The determined MIC levels for silver ion doped HAP–TCP powder was 8 μg/mL for coagulase-negative staphylococcus and 8 μg/mL and 16 μg/mL, respectively, for the comparison of *E. coli* and *S. aureus* [14,15,16,17,18].

### 2.4. Clinical and Radiographic Evaluations

The primary outcome parameter was infection eradication at a minimum of one year after surgery. It was defined as the absence of further surgery performed for infection, no requirement for new systemic antibiotic treatment due to the new onset of symptoms, the absence of recurrent infection with positive cultures from further aspiration or biopsy, and no recurrent sinus formation.

Standard diagnostic imaging modalities, including plain radiography, computed tomography, MRI, and bone scintigraphy, were used to establish the diagnosis. Radiographic evaluation that was used had been established in a previous study by Norden et al. [19], which takes into account the criteria of new periosteal bone formation, presence of sequestrum, bone destruction, and amount of bone involvement, and compared to their follow-up radiographs.

Osseointegration of the synthetic bone graft was also evaluated radiologically.

Complete blood count, C-Reactive Protein, Sedimentation levels, alanine aminotransferase (ALT), aspartate aminotransferase (AST), creatinine, urea levels, and serum and urine silver levels were determined in patients before surgery. Liver and kidney functions were monitored by measuring the same measurements at 2, 4, 6, 10, 14, 21, and 28 days after surgery and then monthly for 1 year. Monthly radiological evaluations were also performed during this period. The duration of the patients’ hospital stay was recorded.

### 2.5. Statistical Analysis

The statistical analysis was performed using IBM SPSS software (version 27.0, Armonk, NY, USA) Statistics. The results are given as the mean, with the standard deviation or range, or as the median and range. Data were tested for normality with the use of the Kolmogorov–Smirnov test. Significance was analyzed with the use of the Student *t* test for unpaired data, with the level of significance set at *p* < 0.05.

## 3. Results

All patients were followed up for 12 months as planned. There were six (50%) female patients and six male patients, and the average age at the time of surgery was 38 years. Study Data for 12 Patients can be found in Table 1.

*S. aureus* was the most abundant microorganism. Eradication of infection was achieved in 11 of 12 patients (91%) as defined by the definition of this study at 12 months. In one patient, the infection was persistent (Patient no. 12, *S. epidermidis*). Except for patient No. 12, all our patients’ infected sinus tracts or wound discharges, if any, have ended. In patient No. 12, wound discharge continued, and then the patient underwent repeated surgeries. The average time to return to normal life functionally varied between 3 and 5 months, and patients were able to put weight on their relevant extremities after the 3rd month. Bone union was observed in all patients except one patient within an average of 3–6 months. No resorption was observed in the grafts at the end of the one-year follow-up. C-reactive protein, sedimentation rate, and leukocyte values decreased significantly in 10 out of 12 patients after the 1-year follow-up (*p* < 0.05). None of the patients had any side effects suggesting silver toxicity, and blood silver level follow-ups were found to be normal. High values of liver function tests Alanine Aminotransferase and Aspartate Aminotransferase (ALT, AST) and kidney function tests (urea, creatinine) were not observed in any patient. When compared with the values of the patients in the control group, no statistically significant difference was found (*p* > 0.05).

### Exemplary Case Presentations

#### Case 1

**Figure 1:**
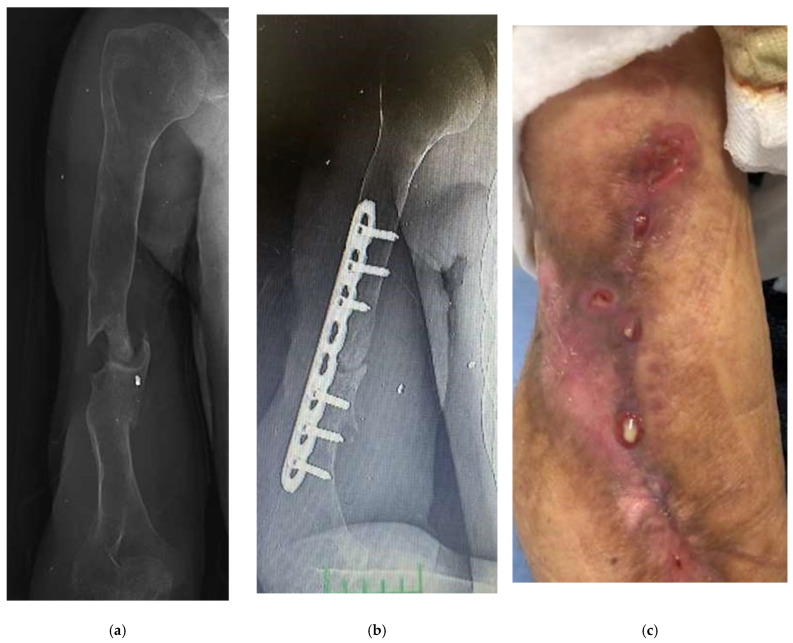

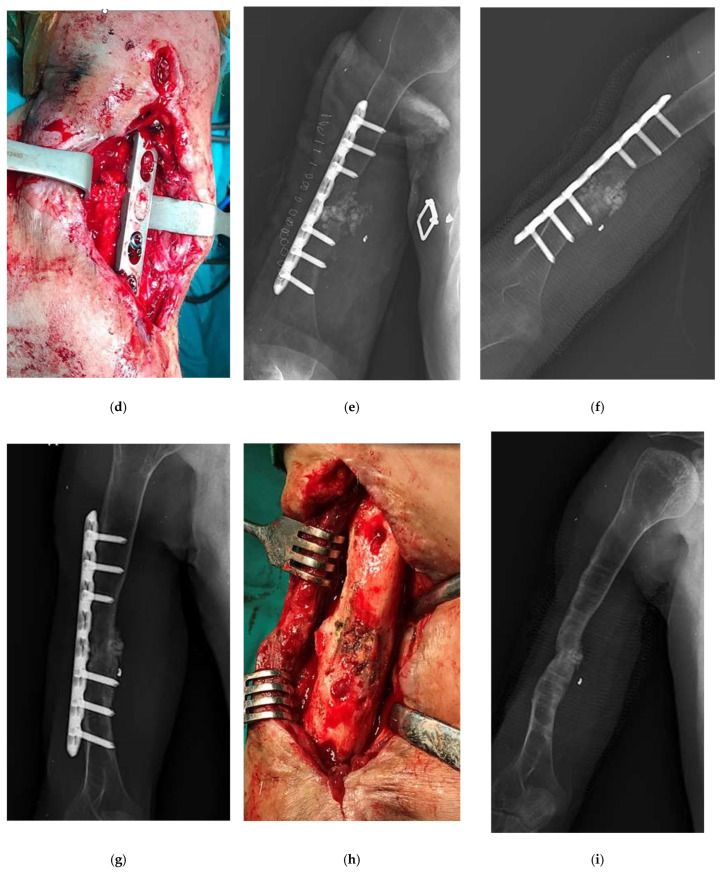
An example case of use of Silver-Doped Synthetic Bone Grafts in A 32-year-old male patient with right humerus osteomyelitis after internal fixation (**a**,**b**) AP radiograph of the right humerus before and after debridement and plate fixation. (**c**) The sinus tract and the extent of infection can be seen on the lateral side of the arm. (**d**,**e**) The plate and screws were not removed, cavity was packed with synthetic bone grafts. (**f**,**g**) The radiograph showed that the graft had adapted to the bone, and later there was bone continuity (**h**) When the implant was removed during surgery, it was visually seen that bone continuity was achieved and the graft was incorporated (**i**) The radiograph showed bone union and consolidation in the second year.

A 32-year-old male patient received external fixator treatment for 9 months due to an open fracture of the right humerus shaft and radial nerve injury after a bomb explosion in the Iraq war. Two years later, an 8-hole plate and autogenous bone grafting were applied in another center with a preliminary diagnosis of humerus pseudoarthrosis (Figure 1a,b). During the debridement of the pseudoarthrosis area, pus was seen, and an intraoperative culture was performed. Pseudomonas aureginosa was grown in the intraoperative culture. Purulent discharge from the wound was observed in the patient who was followed up as an inpatient. The patient, who was given parenteral antibiotics for 2 months, had a sedimentation rate of 42 and a CRP level of 5.9. The patient, who was diagnosed with chronic osteomyelitis and nonunion, presented to us because the discharge from the wound continued, and the infection parameters did not regress (Figure 1c).

The patient underwent fistulography during surgery. It was observed that the methylene blue extended to the sequestra. The sequestra were removed, and bone ends were debrided, continuing until healthy, bleeding bone was exposed. The area was then irrigated extensively, and the cavity was packed with synthetic bone grafts (Figure 1d,e). The intraoperative tissue sample sent from the patient was reported to be chronic osteomyelitis. Staph aureus grew in the patient. The sedimentation rate, which was 90, and the CRP, which was 32 the week after surgery, became 28 and 1.7, respectively, 6 weeks later. The patient, who came to his follow-ups regularly for the first 3 months, did not come to his follow-ups later because he was sent to prison. There was no discharge in the wound area in the examination of the patient who came 18 months after surgery. The leukocyte count was 8000, the sedimentation rate was 20, and the CRP was 1.6. The X-rays showed that bone healing, the graft had adapted to the bone, and there was bone continuity (Figure 1f,g). 19 months after the operation, it was requested that the plate and screw be removed because the skin was thin and the metal implant was felt under the skin, causing extreme discomfort to the patient. When the implant was removed during surgery, it was visually seen that bone continuity was achieved and the graft was incorporated (Figure 1h). At the second year postoperative period, it was determined that the patient returned to his daily life, radiologically, his bone integrity was ensured, and he was able to use his arm (Figure 1i).

## 4. Discussion

The most important findings of this study were that the silver ion-doped calcium phosphate-based synthetic bone graft material, as an antimicrobial carrier used in patients with chronic osteomyelitis, eradicated infection in 11 of 12 patients at twelve months. No resorption was observed in the grafts at the end of the one-year follow-up, and none of the patients had any side effects suggesting silver toxicity. This result is superior to the antibiotic-eluting, resorbable, calcium-based scaffolds used for chronic osteomyelitis published in prior reports [20].

Given the increasing number of surgeries performed each year and the aging population, the absolute number of implants used in the treatment of musculoskeletal disorders and related infections is expected to continue to increase. Unfortunately, decades of research and improvements in clinical care, infection prevention protocols, perioperative antibiotics, and aseptic surgical techniques have not prevented the high incidence of bone infections. Successful treatment requires adequate surgical debridement with wide excision, together with long-term, high antibiotic concentrations that are best achieved by local delivery devices, either made of degradable or nondegradable materials. For several years, bone defects in osteomyelitis have been treated through debridement in combination with gentamicin-loaded polymethyl methacrylate (PMMA). However, this method is not free of drawbacks, such as thermal damage to the antibiotic, the need for a second intervention to remove PMMA, and facilitation of biofilm formation by multiresistant colonies [5,21]. The use of antibiotic-impregnated bone allografts and antibiotic-eluting, biodegradable, calcium-based scaffolds in the treatment of chronic osteomyelitis has also not been sufficient to solve the problem due to reasons such as short-term effectiveness and antibiotic resistance [6,22,23].

Current research on this subject was directed towards the development of new biomaterial systems with antimicrobial properties. The greatest success will be in the development of biomaterial systems that will eliminate infection while contributing to bone regeneration [24,25]. The ideal antimicrobial agent for bone infections should be able to reach high local concentrations without causing systemic toxicity. It should also be effective against sessile forms of bacteria and should not disrupt bone regeneration and the integration of the implant with biological tissues. Global rise in antibiotic resistance poses a significant threat, diminishing the efficacy of common antibiotics against widespread bacterial infections. World Health Organization (WHO) declared that Antimicrobial resistance is one of the top global public health threats. It affects countries in all regions and at all income levels. It is estimated that bacterial resistance was directly responsible for 1.2 million global deaths in 2019 and contributed to 5 million deaths [26,27].

The gold standard for diagnosing bone infection has traditionally been the isolation of a microorganism from microbiological cultures. However, despite clinical evidence confirming the presence of infection, this may not always be possible (culture-negative infections). The prevalence of culture-negative infections has been reported to be as high as 40% [28]. Possible reasons for negative cultures include infection by fastidious pathogens, biofilm encapsulation, rare organisms that do not grow in routine culture media, and inadequate sampling or transport. However, the most important reason for failure to isolate an organism is the administration of antibiotics before collecting samples from the infection site. Despite negative cultures in two of our patients, we continued treatment because the clinical presentation, blood, and radiographic examinations confirmed the presence of bone infection. Staphylococcus aureus was the most common microorganism in the patients included in the study. It is known in the literature that Staphylococcus aureus causes approximately 70% of bone and joint infections and is resistant to treatment due to biofilm formation. In addition, *S. aureus* has been reported to infiltrate and persist in many host cell types, including osteocytes, osteoblasts, osteoclasts, synovial fibroblasts, and synovial macrophages in the musculoskeletal environment, resulting in the development of recurrent osteomyelitis in patients years after the index infection [29,30,31].

After debridement of chronic osteomyelitis, the ideal dead space management strategy is crucial to eliminate microorganisms in the environment. The space-filling material should not adversely affect healing on local bone tissue, should be systemically and locally nontoxic, should have a predictable resorption rate, should be able to act as an antimicrobial agent or carrier, and should be gradually replaced by the host bone. Bacterial intracellular persistence is one of the reasons why these space-filling agent strategies are not completely successful in treating *S. aureus* bone and joint infections [32]. The high success rate of the antimicrobial bone substitute used in this study in eliminating foci of infection may be attributed to its long-term antimicrobial effect on staph aureus during its gradual resorption. There is no elution of antimicrobial substances into the environment. As the synthetic bone graft becomes incorporated with the bone tissue, silver ions emerge in the contact surface areas and show their antimicrobial effect. Thus, the antimicrobial effect will continue until the bone graft applied to the resulting gap is completely incorporated. The system may be complementary to systemic therapy in the treatment of osteomyelitis because it fulfills all the required properties that a carrier should have, such as tissue compatibility and sustained release of high antimicrobial concentrations.

The increase in antibiotic-resistant pathogens leads to consideration of a material-based view of antimicrobials for osteomyelitis [33]. The antimicrobial properties of silver are well-documented, and silver is integrated into many types of medical devices, including catheters, vascular prostheses, bandages, and orthopedic implants. The clinical incidence of silver-resistant bacteria remains low because silver, unlike common antibiotics, activates multiple mechanisms and hits different targets within the bacterial cells [34]. The silver toxicity is correlated to its bioavailability and determined by its solubility, oxidation state, complexation ability toward biological targets, excretion, and detoxification routes. Not all silver is the same. In medicine and orthopedics, silver is used for its antimicrobial effect in various forms, including metallic silver, ionic silver, and nanoparticle silver, and in different dosages. Consequently, conflicting publications regarding the antimicrobial activity and toxicity of silver can be found in the literature. Guo et al. [34] measured the levels of free/reactive oxygen radicals in vascular endothelial cells in an animal study they published. The study showed that by increasing the levels of oxygen radicals in vascular endothelial cells, nanocomposite particles, rather than intravenously injected silver ions, caused long-term organ toxicity. Therefore, when evaluating the toxicity and efficacy of silver, the total amount of silver used in the patient and the form of silver should be considered. In our previous publications, we have shown that the coating we developed prevents infection, does not disrupt angiogenesis, which plays an important role in bone tissue growth, and is not cytotoxic at the concentration used [14]. Similarly, a recent study has shown that ionic silver coating of orthopedic implants does not impair osteogenic differentiation and mineralization at Ag^+^ concentrations up to 0.5 ppm [35]. Although the small number of cases is an important limitation, it was found acceptable because of the use of a new biomaterial for treatment. The second important limitation is the short follow-up period.

The use of silver in the treatment and prevention of infection is widespread in the medical community today. Advances in technology have enabled its use to be incorporated into orthopedic practice with increasing success. When silver-ion is used together with a bioactive material such as hydroxyapatite and calcium phosphate, which are synthetic bone substitutes, a material with high osteointegration and antimicrobial activity is obtained. In patients with chronic osteomyelitis, synthetic bone substitutes doped with silver ions can be used instead of acrylic cement to avoid reoperation for cement removal. The excellent pharmacologic and biomechanical behavior of the local chemotherapy system we developed needs additional testing in well-designed experimental and clinical trials for the treatment of chronic osteomyelitis.

## 5. Conclusions

This retrospective study on silver ion-doped calcium phosphate synthetic bone grafts in 12 patients with chronic osteomyelitis, infected non-unions, or implant-related infections demonstrates highly encouraging results that could significantly influence the management of difficult chronic bone infections. With this method, 91.7% infection eradication was achieved in 12 months, bone union was achieved in 11 of 12 patients within 6 months, and graft resorption and systemic silver toxicity were not observed.

Key implications

- Enables single-stage surgery with simultaneous dead-space management and prolonged local antimicrobial delivery.
- Reduces need for prolonged systemic antibiotics, multiple revisions, or antibiotic-loaded spacers.
- Potential paradigm shift in orthopedics, trauma, spine, and prosthetic joint infection management—especially valuable in antibiotic-resistant era and resource-limited settings.
- If confirmed in larger trials, could become a cornerstone treatment for chronic bone infections, offering a safe, off-the-shelf alternative to autologous grafting and traditional local antibiotic carriers.

## Figures and Tables

**Table 1:** Study Data for 12 Patients.

No.	Sex	Age	Cause	Clinic	Diagnosis	CRP First/Last	Sedim First/Last	Microorganism	Result
1	M	32	In-car traffic accident	Discharge in the right thigh, pain, inability to step on it, increased temperature	Posttraumatic osteomyelitis of femur	4.9 1.4	26 7	Polymicrobial	Healed
2	M	34	Fracture Related Infection	Pain, tenderness, increased temperature, loss of function, discharge in the finger in the 2nd year after the fracture	Infected proximal phalanx	21.8 12	39 10	Polymicrobial	Healed
3	F	46	In-car traffic accident	Pain, tenderness, increased temperature, and loss of function in the right arm at 2 years after a right humerus shaft fracture	Chronic osteomyelitis of humerus	4.2 4.1	19 16	Culture-negative	Healed
4	M	29	Hand crush, work accident	Metatarsal discharge, pain, tenderness, increased temperature, loss of function	Posttraumatic Chronic osteomyelitis of metacarpal.	2.9 1.6	12 3	Polymicrobial	Healed
5	M	32	Humerus open fracture, radial nerve injury	Open humerus fracture treated with external fixator, discharge pain, 9th month postoperatively tenderness, increased temperature, loss of function and nonunion in the	Infected non-union of humerus	14 2	42 17	Polymicrobial	Healed
6	M	24	Fracture Related Infection	Surgery for pelvic fractures + fracture dislocation in the left ankle falling from the height	Posttraumatic tibia osteomyelitis	10.7 4.4	39 8	Single organism	Healed
7	F	34	Calcaneal osteomyelitis in a gangrenosum patient with pyoderma	Discharge in the right heel, redness, tenderness, inability to bear weight, loss of function	Chronic osteomyelitis of calcaneus	7.6 0.9	127 79	Polymicrobial	Healed
8	F	48	Right knee pain.	Right leg pain, swelling, tenderness, difficulty walking	Chronic osteomyelitis of tibia	5.2 3.7	22 15	Culture-negative	Healed
9	F	36	Right knee pain.	Pain, swelling, tenderness, difficulty walking in the right leg	Chronic osteomyelitis of tibia	122 0.7	101 23	Single organism	Healed
10	M	65	Fracture Related Infection	the skin, difficulty in walking. Increased temperature at the wound site, redness, fistulized discharge to	Posttraumatic tibia osteomyelitis	27.4 7.1	58 8.3	Single organism	Healed
11	F	44	Fracture Related Infection	Increased temperature, redness, fistulized discharge to the skin, difficulty in walking	Posttraumatic tibia osteomyelitis	1.2 2.3	16 14	Single organism	Healed
12	F	37	After suicide attempt Fracture Related Infection	double fracture + distal tibia + left medial malleolus fractures Right acetabulum anterior column + right sacrum + left anterior arm Increased temperature, redness, fistulized discharge to the skin, difficulty in walking	Posttraumatic tibia osteomyelitis	21.8 9.8	37 54	Polymicrobial	Poor

## Data Availability

The data supporting the findings of this study are not publicly available due to ethical and privacy restrictions. De-identified data may be provided by the corresponding author upon reasonable request and with permission of the Eskisehir Osmangazi University Ethics Committee.
